# Roles of NMDA and dopamine in food-foraging decision-making strategies of rats in the social setting

**DOI:** 10.1186/s12868-015-0233-8

**Published:** 2016-01-11

**Authors:** Fang Li, Wen-Yu Cao, Fu-Lian Huang, Wen-Jing Kang, Xiao-Lin Zhong, Zhao-Lan Hu, Hong-Tao Wang, Juan Zhang, Jian-Yi Zhang, Ru-Ping Dai, Xin-Fu Zhou, Chang-Qi Li

**Affiliations:** Department of Anatomy and Neurobiology, School of Basic Medical Science, Central South University, Tongzipo Road 172, Changsha, 410013 Hunan China; Department of Anesthesia, the Second XiangYa Hospital, Central South University, Ren-Min Road 86, Changsha, Hunan China; School of Pharmacy and Medical Sciences, Sansom Institute, University of South Australia, Adelaide, SA Australia

**Keywords:** Food-foraging behavior, Social decision-making, Mimetic desire, Rats

## Abstract

**Background:**

In highly complex social settings, an animal’s motivational drive to pursue an object depends not only on the intrinsic properties of the object, but also on whether the decision-making animal perceives an object as being the most desirable among others. Mimetic desire refers to a subject’s preference for objects already possessed by another subject. To date, there are no appropriate animal models for studying whether mimetic desire is at play in guiding the decision-making process. Furthermore, the neuropharmacological bases of decision-making processes are not well understood. In this study, we used an animal model (rat) to investigate a novel food-foraging paradigm for decision-making, with or without a mimetic desire paradigm.

**Results:**

Faced with the choice of foraging in a competitive environment, rats preferred foraging for the desirable object, indicating the rats’ ability for decision-making. Notably, treatment with the non-competitive N-methyl-d-aspartate receptor antagonist MK-801, but not with the dopamine D1 or D2 receptor antagonists, SCH23390 and haloperidol, respectively, suppressed the food foraging preference when there was a competing resident rat in the cage. None of these three antagonists affected the food-foraging preference for palatable food. Moreover, MK-801 and SCH23390, but not haloperidol, were able to abolish the desirable environment effect on standard food-foraging activities in complex social settings.

**Conclusions:**

These results highlight the concept that mimetic desire exerts a powerful influence on food-foraging decision-making in rats and, further, illustrate the various roles of the glutamatergic and dopaminergic systems in mediating these processes.

## Background

Because rodents are highly social animals, many of their important social decisions, including choice of mate and food-foraging, are made within the social setting [1, 2]. Social decision-making, an enormously complex cognitive function, is typically made by cooperation with and conflict among conspecifics [3, 4]. In social psychology, the mimetic desire phenomenon, recognized as social contagion and a widespread strategy in nature, is one in which an object belonging to one person tends to become a goal for the observer. In humans, mimetic desire is able to effectively influence pursuit of an object [5]. In addition to competitive food-snatching from other animals in the social setting, food-foraging decision-making is a process modulated by environmental factors and/or nutritional needs [6–9]. Due to the complexity of these processes, both the required interactive scenario, along with straightforward experimental manipulations, present challenges in the controlled laboratory setting. Our previous investigations successfully created an ideal food-foraging animal (rat) model capable of quantitatively demonstrating natural food-foraging behavior without either artificial interventions or training [10]. We found that food-foraging is associated not only with the choices animals make but also with higher cognitive functions [11, 12].

A growing body of studies in behavioral economics have used non-invasive neuroimaging techniques, such as functional magnetic resonance imaging (fMRI) and magnetoencephalography, and computational approaches to investigate the neural mechanisms behind sophisticated decision-making strategies [13–16]. A recent fMRI study reported that the human anterior cingulate cortex (ACC) is able to encode environmental signals as reflecting estimates of the richness and cost of the foraging environment [17].

Many psychiatric disorders involve deficits in decision-making, as well as dysregulated dopamine and/or dopamine receptor expressions [18, 19]. Additional studies have suggested that dopamine and/or dopamine receptors play vital roles in modulating the balance between effort and benefit, and goal-directed action selection, encoding the differences between actual and expected rewards [20, 21]. Dopamine receptors interact strongly with N-methyl-d-aspartate (NMDA) receptors, and the activities of each receptor mediates dissociably cost/benefit decision-making processes by means of effort- or delay-discounting procedures [22, 23]. Using the Iowa Gambling Task (IGT), Ness et al. found that polymorphisms of the NMDA receptor 2B subunit gene influence decision-making [24]. Low-impulsive rats showed a greater preference for immediate and smaller rewards after ketamine or memantine manipulation [25].

In the present study, we developed a model for testing decision-making in both desirable and non-desirable environments, and examined the notion that social-based decisions in food-foraging contexts may be differentially mediated by NMDA versus dopamine receptors.

## Methods

### Animals

Male Sprague–Dawley (SD) rats (inbred strain), each weighing 250–300 g at the beginning of the experiment, were obtained from the Animal Center of Central South University. Animals were housed in a temperature (23 ± 2 °C) and humidity (50 ± 5 %) controlled animal facility. All experimental rats were housed together in 50 × 35 × 20 cm cages (n = 3/cage) and maintained on a 12-h light/dark cycle with free access to food and water. All animals were adapted to laboratory conditions for 1 week prior to experimental manipulations. The experimental protocol was approved by the Animal Care and Use Committee of Central South University and conformed to the National Institutes of Health Guide for the Care and Use of Laboratory Animals. All efforts were made to minimize the number of rats used, as well as any suffering. Each treatment group comprised 8–16 rats. We tested several different procedures, but no animal experienced more than one procedure/session. Rats were randomly assigned to experimental procedures, as described below.

### Behavioral procedures

**Fig. 1:**
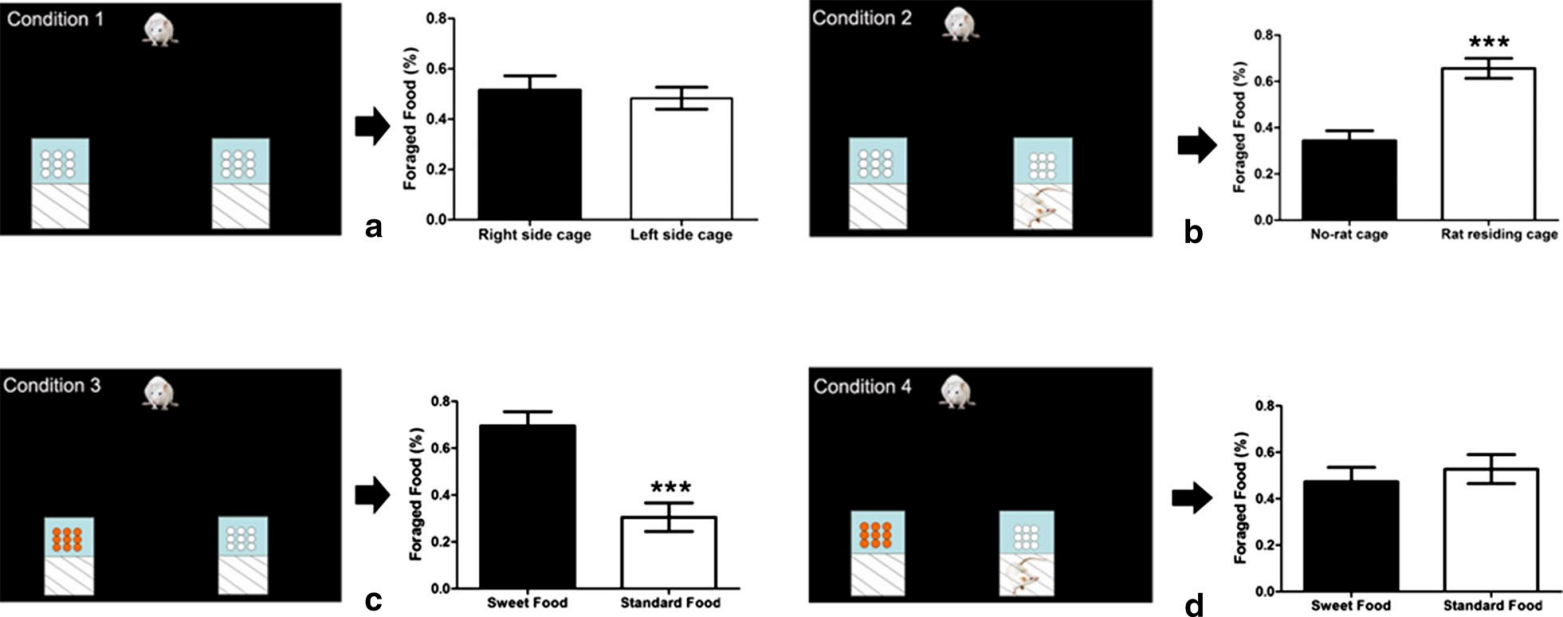
Schematic photographs of the decision-making paradigm designs and performances with or without social information. Two small wire-topped plastic home cages (30 × 18 × 16 cm) with 200 g standard rodent food pellets on both side of cages, **a**, **b** (*white*); *right* side cage, **c**, **d** (*white*); or sweet food on *left* side cage, **c**, **d** (*red*) on the removable wire mesh placed in the open field. A rat of the same gender resided on the *right* side of cage for at least 1 week before the experiment (**b, d**). For each trial, the open-field rat had to make a choice and select to forage food from either or both of the two cages. **a** Student^’^s t- test indicated that there was no significant difference in the ratio of foraged standard food from either side of cages; **b** the percentage of foraged standard food pellets was increased significantly from the rat-residing cage relative to that from the no-rat cage; **c** rats preferred to forage sweet food pellets. The percentage of foraged standard food pellets was less than that of foraged sweet food pellets; **d** there was no significant difference between the percentage of foraged standard food pellets from the rat-residing cage and foraged sweet food pellets from the no-rat cage. ***p<0.001 represents statistically significant differences compared to foraged standard food pellets from the no rat cage (**b**), ***p<0.001 represents statistically significant differences compared to foraged sweet food (**c**)

Each test rat was placed in a sound-attenuating environment consisting of an open-field apparatus (150 × 150 × 50 cm) constructed of black wood. Procedures were conducted as previously reported, with several modifications [10, 12]. Food pellets (200 g) were placed in two small plastic cages (30 ×18 × 16 cm), each equipped with a removable metal wire lid. On test days, test rats were removed from their home cages, placed in the open-field apparatus, and allowed to acclimate for 2 h, after which one of the following conditions was randomly applied: (Fig. 1).

#### Condition 1

No rats in cages; access to standard food pellets on the removable wire lid (Fig. 1a).

#### Condition 2

Two cages with standard food pellets on the removable wire lid. Two rats of the same gender, one in a cage and the other in the open field (Fig. 1b).

#### Condition 3

No rats in either cage, with standard food pellets on the removable wire lid of one cage and sweet (10 % sugar) food pellets on the lid of the other cage (Fig. 1c).

#### Condition 4

One rat in cage with standard food pellets, and one cage with sweet food pellets and one other rat of same gender in the open field (Fig. 1d).

The test rat in the open field was allowed to navigate freely to the cages and forage the food pellets from 7:00 to 9:00 pm. The quantity of food pellets moved to the open field and the quantity of food pellets left in the food containers were each determined at 9:00 pm. In these experiments, the quantity of foraged food was calculated by the formula: 400 g minus the amount left in both food containers. The amount of food eaten was calculated by the formula: 400 g minus the amount of food pellets moved to the open field minus the amount left in both food containers. The percentage of foraged food pellets from one food container was calculated by the formula: the amount of foraged food divided by the difference between 200 g and the amount left in the food container during the 2-hour test. Two removable wire lids were placed in the rat-containing cage to isolate the rat and food pellets in the container under Conditions 2 and 4, such that the rat in the cage has no access to the food on top of the cage.

### Effects of social information on food-foraging decision-making after pharmacological manipulations

To determine whether NMDA- and dopamine receptors -modulated social information in food-foraging decision-making is dose-dependent, we tested various dosages of MK-801, SCH23390, and haloperidol, according to previous studies, but with several modifications [10, 26–30]. MK-801 (0.05, 0.1, 0.15, or 0.2 mg/kg, catalog number: M107), SCH 23390 (0.005, 0.015, or 0.025 mg/kg, catalog number: D054), or haloperidol (0.01, 0.015, 0.02, or 0.05 mg/kg, catalog number: H1512) were administered intraperitoneally and represented NMDA, D1-like, and D2-like receptor antagonists, respectively, for each task. All drugs were purchased from Sigma (Sigma-Aldrich, St. Louis, MO, USA) and were dissolved (with the exception of haloperidol) in saline. Haloperidol was dissolved in 10 % glacial acetic acid, brought to volume with saline, and pH-adjusted to 6.5 with NaOH. Sterile 0.9 % saline was used as the vehicle. Rats were assigned randomly to different drugs and dosage groups, with each rat receiving only one injection of vehicle or drug. For each drug tested, rats were allowed to perform decision-making tasks 30 min after treatment with drug or vehicle (Conditions 1–4).

### Statistical analyses

Statistical analyses were performed using version 13.0 SPSS software (SPSS Inc., Chicago, IL, USA) and Prism 5.0 software (Graphpad Software, San Diego, CA, USA). Results are presented as mean ± SEM. Statistical differences in behavioral results were determined using one- or two-way analysis of variance (ANOVA), followed by post hoc Dunnet’s test, Tukey post hoc multiple comparison test, or Bonferroni post-test, where appropriate. Unpaired two-tailed Student’s *t* test was used if only two groups were applied. Differences were considered significant when the *p*-value <0.05.

## Results

### Social influence on food-foraging decision-making

As seen in Fig. 1, rats were able to freely forage food pellets from two food containers to the field under all conditions (1–4). The percentages of foraged standard food pellets from the right and left cage were 51.48 ± 5.74 and 48.33 ± 4.39 %, respectively. No significant difference was observed between these cages under Condition 1 (t = 0.436, *p* = 0.669, n = 9) (Fig. 1a). Conversely, under Condition 2, the test rat in the open field in the company of a conspecific showed a preference for foraging standard food pellets from the rat-residing cage (65.62 ± 4.34 %) compared to the cage with no rat (34.38 ± 4.34 %, t = −5.085, *p* < 0.001, n = 9) (Fig. 1b). Under Condition 3, the percentage of foraged standard food pellets (30.53 ± 6.08 %) was lower than that of foraged sweet food pellets (69.47 ± 6.09 %, t = 4.522, *p* < 0.001, n = 12) (Fig. 1c). However, under Condition 4, no significant difference between foraged sweet (47.28 ± 6.23 %) and foraged standard (52.72 ± 6.23 %) food pellets was observed (t = −0.617, *p* = 0.547, n = 8) (Fig. 1d).

### NMDA and DA antagonists in modulating food-foraging decision-making

Under Condition 2, control rats with injected vehicle and experimental rats with various doses of haloperidol, MK-801, or SCH 23390 were tested for food-foraging behaviors.

**Fig. 2:**
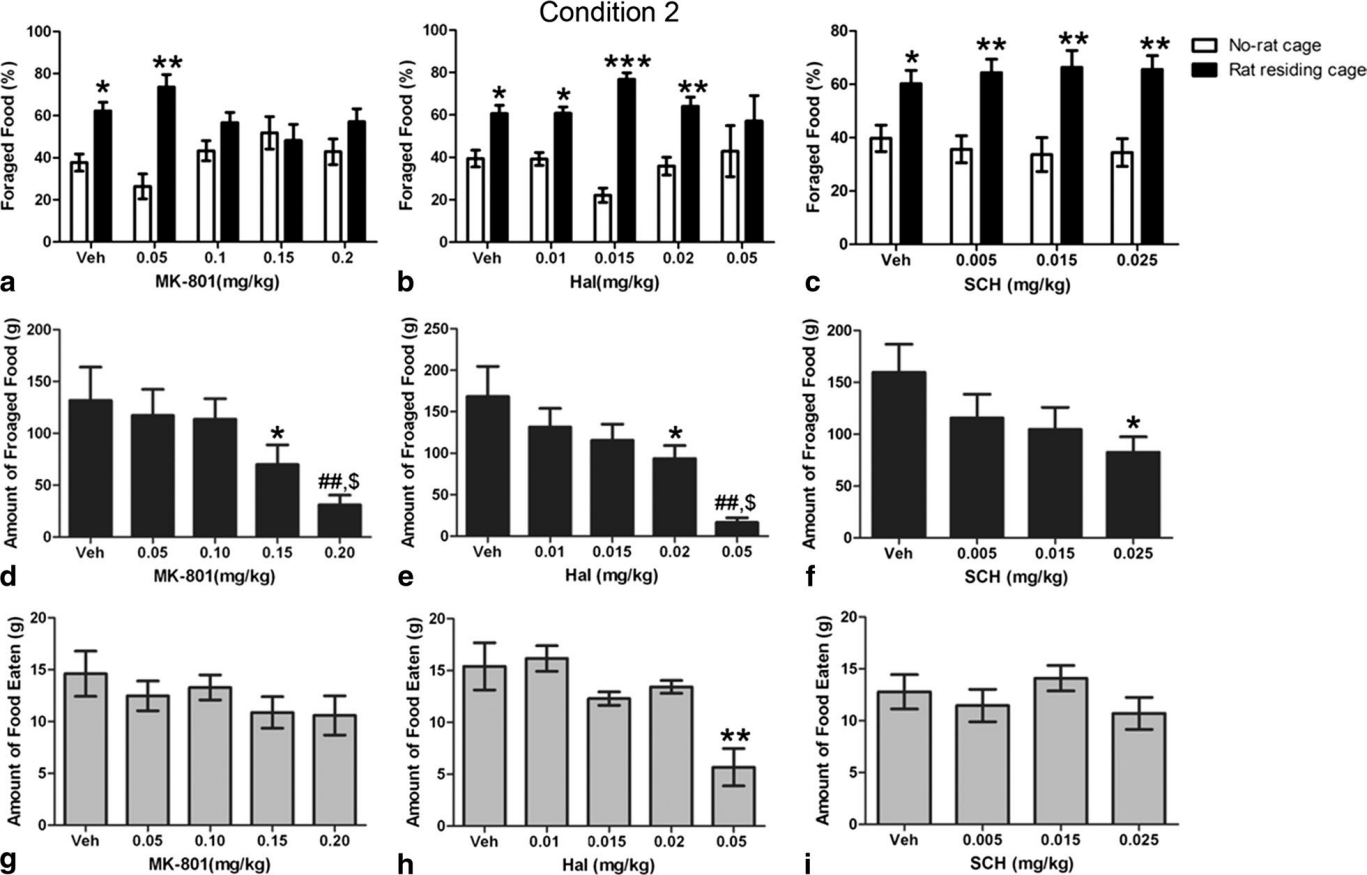
Effects of different dosages of MK-801, haloperidol, and SCH23390 on the percentage of foraged standard food pellets (**a**–**c**), amount of foraged food (**d**–**f**), and amount of eaten food (**g**–**i**) under Condition *2*. **a** There are no differences in the ratio of foraged standard food pellets from the cage with or without a residing rat after administration of 0.1, 0.15, or 0.2 mg/kg MK-801; **b**–**c**. the percentage of foraged standard food pellets from the rat-residing cage in vehicle, haloperidol, and SCH23390 treatment groups were significantly increased compared with the no-rat cage. *p<0.05, **p<0.01, ***p<0.001 represent statistically significant differences compared to foraged standard food pellets from the no-rat cage; **d**–**f**. The total amount of foraged food decreased with increasing dosages of MK-801, haloperidol, and SCH 23390 treatment. **d** *p<0.05 vs veh group, ##*p*<0.01 vs veh, 0.05 mg/kg and 0.10 mg/kg group, $*p*<0.05 vs 0.15 mg/kg group; **e** *p<0.05 vs veh group, ##*p*<0.01 vs veh group, $*p*<0.05 vs 0.01 mg/kg group; **f** *p<0.05 vs veh group; **g**–**i** no obvious alterations of the amount of food eaten were observed after MK-801 or SCH 23390 treatment; The amount of food eaten decreased after 0.05 mg/kg haloperidol injection. **p<0.01 vs the other group

After MK-801 treatment, there was a dose-dependent effect on the percentage of foraged food. The quantity of foraged food in the cage with a resident rat increased significantly (t = 2.700, *p* < 0.05, n = 10) after vehicle treatment. MK-801, at a dosage of 0.05 mg/kg, had no effect on the amount of foraged food, i.e., the amount of foraged food from the cage with a resident rat had still increased (t = 4.639, *p* < 0.01, n = 8), with no difference compared with control rats. In contrast, MK-801, at the higher doses of 0.1 mg/kg (t = 1.866, *p* > 0.05, n = 16), 0.15 mg/kg (t = 0.4192, *p* > 0.05, n = 11), and 0.2 mg/kg (t = 1.858, *p* > 0.05, n = 14) suppressed the preference of food-foraging completely when there was a competing rat in the cage. These results suggest that glutamatergic neurotransmission is involved in the food-foraging decision-making process in the social environment. One-way ANOVA indicated that the total amount of foraged food decreased in parallel with increased in MK-801 dosage[F_(4,58)_ = 4.173, *p* < 0.01, n = 8–14] (Fig. 2d). However, no obvious alterations in the amount of food eaten occurred after treatment with various dosages of MK-801 [F_(4,58)_ = 0.994, *p* > 0.05, n = 8–14] (Fig. 2g).

Low dosage (0.01–0.02 mg/kg) treatment with haloperidol did not affect the decision-making process on the preference for foraging from the social environment, as the percentage of foraged food from the cage with a resident rat had still increased [F_(4,78)_ = 0.003, *p* < 0.05, n = 8–10]. However, a higher (0.05 mg/kg) dosage abolished this preference, as there was no difference in the amount of foraged food between the two cages (t = 1.623, *p* > 0.05, n = 8, Fig. 2b). One-way ANOVA showed that the total amount of foraged food (Fig. 2e) [F_(4,43)_ = 3.189, *p* < 0.05, n = 8–10] and the amount of eaten food [F_(4,43)_ = 7.929, *p* < 0.001, n = 8–10] had decreased with increased haloperidol dosages (Fig. 2h).

Treatment with SCH 23390 did not alter the preference for social-environment food-foraging. Compared with vehicle-treated rats, there was no difference in the percentage of food foraged from the rat-residing cage after SCH 23390 treatment at all dosages [F_(3,31)_ = 0.248, *p* > 0.05, n = 8] (Fig. 2c). One-way ANOVA indicated that the total amount of foraged food decreased with increasing dosages of SCH 23390 [F_(3,32)_ = 2.949, *p* < 0.05, n = 8] (Fig. 2f), but there were no outstanding changes in the amount of food eaten after SCH 23390 treatment [F_(3,32)_ = 2.029, *p* > 0.05, n = 8] (Fig. 2i).

**Fig. 3:**
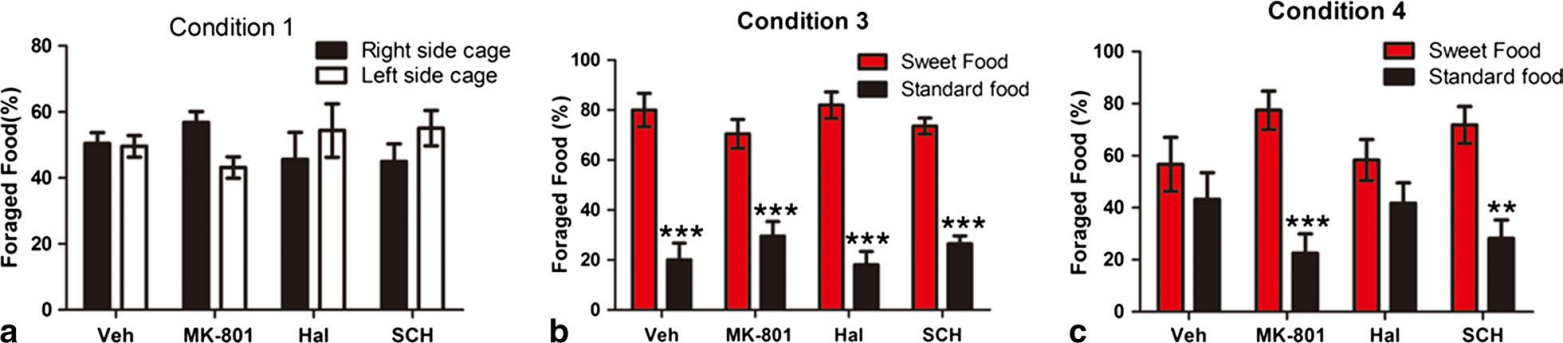
Effects of MK-801, haloperidol, and SCH 23390 on the percentage of foraged food pellets under Conditions *1*, *3*, and *4*. **a** Bonferroni post-tests revealed no significant effects of vehicle, MK-801, haloperidol, or SCH 23390 treatment on the percentage of foraged food from both side cages under Condition *1*; **b** rats preferred foraging sweet food pellets to standard food pellets among the four groups under Condition *3*. Bonferroni post-tests revealed significant effects of vehicle, MK-801, haloperidol, and SCH 23390 treatment on the percentage of foraged of sweet food pellets compared with standard food pellets. ***p<0.001, represents statistically significant differences compared with foraged sweet food pellets; **c** when the test rat confronted a rat in one cage with standard food pellets, the percentage of foraged standard food pellets after MK-801 and SCH23390 treatment decreased significantly compared with foraged sweet food pellets in the other cage. No distinct differences between the two kinds of food pellets were observed after vehicle or haloperidol treatment under Condition *4*. **p<0.01, ***p<0.001 represents statistically significant differences compared with foraged sweet food pellets

We selected 0.1 mg/kg MK-801, 0.015 mg/kg haloperidol, and 0.015 mg/kg SCH23390 for the other three testing sessions. As shown in Fig. 3a, no primary effects were associated with side-of-cage [F_(1,50)_ = 0.0768, *p* > 0.05, n = 7–8], drug treatment [F_(3,50)_ = 0.0000, *p* > 0.05, n = 7–8], or their interactions [F_(3,50)_ = 0.0000, *p* > 0.05, n = 7–8] on the percentage of food foraged under Condition 1. Bonferroni post-tests revealed no dramatic effects of vehicle, MK-801, haloperidol, or SCH 23390 treatments on the percentage of food foraged from either side of cages (*p* > 0.05, n = 7–8).

Under Condition 3, dissimilar foods (standard or sweet) affected the percentage of food foraged (Fig. 3b) [F_(1,54)_ = 183.6, *p* < 0.0001, n = 7–10], Drug treatment had no major contributing effect [F_(3,54)_ = 0.0000, *p* > 0.05, n = 7–10], nor a significant interaction [F_(3,54)_ = 0.0000, *p* > 0.05, n = 7–10], on foraged food preference. These results suggest that rats treated with MK-801, haloperidol, or SCH 23390 were not affected significantly in their preference for sweet food foraging.

Under Condition 4, two-way ANOVAs indicated that the percentage of foraged food (Fig. 3c) was affected by food type (standard or sweet) [F_(1,60)_ = 28.56, *p* < 0.0001, n = 8–11] as well as an interaction between food type and the drug used [F_(3,60)_ = 2.897, *p* < 0.05, n = 8–11]. Similar to unmanipulated condition, vehicle treatment in the presence of a conspecific led to no significant differences in foraging from standard chow or palatable food (t = 1.100, *p* > 0.05, n = 8) (Fig. 3c). Treatment with either MK-801 (t = 4.560, *p* < 0.001, n = 8) or SCH23390 (t = 3.573, *p* < 0.01, n = 8), but not haloperidol (t = 1.621, *p* > 0.05, n = 11), significantly decreased the percentage of foraged standard food pellets compared with foraged sweet food pellets in the other cage when the test rat confronted a rat in one cage with standard food pellets (Fig. 3c).

## Discussion

Extrapolating food-foraging decision-making behaviors of animals in the natural social-setting to the laboratory-setting requires an understanding of the specific neurobiological and pharmacological bases responsible for integrating complex social interactions. In humans, mimetic desire as a socially meaningful stimuli, has a great impact on social interactions in everyday adult life [31]. Despite broad interest in the neural mechanisms involved in decision-making, little is known about the effects of mimetic desire on decision-making and its underlying mechanism(s). In our double-blind, placebo-controlled study, we (1) used different food-foraging contexts to determine how mimetic desire is involved in foraging food from cages with another conspecific; (2) investigated the role(s) of glutamate and dopamine receptors in modulating mimetic desire in the decision-making process; and (3) uncovered various neural substrates associated with food-foraging decision-making in the social setting. Our multi-perspective approach allowed for a greater understanding of animal social decision-making because it included a variety of food-foraging tasks, in combination with pharmacological manipulation, that compelled the animal to make different choices.

Notably, the ratio of foraged standard food pellets from the rat cage-residing to that of the open-field rat was significantly greater under Condition 2. We speculate that the open-field rat was able not only to interact with the cage rat by means of olfactory, acoustic, and non-contact visual cues, but also viewed the food pellets from the cage-residing rat as more “likable” objects than those from no rat residing cage. In social perception and behaviours, mimetic desire, represents a special case of evaluative conditioning, is examined by target’s facial trustworthiness paradigm [32]. The vicarious observation of the outcomes of others can guide action options, resulting in the adoption of advantageous behavioral traits and adaptation to changing environments [33]. Interestingly, the decision for preferentially foraging food from a socially desirable environment was disrupted by manipulation of MK-801, but not SCH23390 or haloperidol, indicating that NMDA receptors, but not dopamine receptors, are required for regulating food-foraging decision-making processes within the social context. In our previous study, treatment with MK-801 reduced competitive food foraging, but did not affect the food foraging behavior in non-competitive and no-hurdle food foraging tests, suggesting that the NMDA receptor may be involved in regulating competitive activity [10]. The residing rat in the small home cage can interact with the test rat, which was defined as a potential competitor and sought to forage food from the wire mesh under Condition 2. We presume that the observed decrease in the amount of foraged food in a socially competitive environment after MK-801 administration (i.e., 0.1, 0.15, or 0.2 mg/kg) could have been due to blockade of NMDA receptor, resulting in disruption of competitive activity and disrupting the modulation dynamics of a socially competitive environment on food foraging decision making. We observed that 0.02 and 0.05 mg/kg haloperidol treatment decreased the total amount of foraged food, which is consistent with our prior observation of a dramatic decrease in foraged food by rats after higher-dose haloperidol (0.1 mg/kg) in the competitive, non-competitive, and no-hurdle food foraging tests [10]. A limitation of the current research is that we have not delineated the precise mechanism by which the glutamatergic system influences the process. In this regard, it is interesting to note that glutamate in the perigenual ACC plays a vital role in regulating impulsiveness and risk probability during decision-making in the Cambridge Gambling Task [34]. Frye et al. showed that glutamate levels in the anterior cingulate/medial prefrontal cortex was significantly elevated in bipolar depressed subjects with maladaptive decision making behavior relative to that in healthy controls [35]. Hoerst et al. observed a correlation between glutamate concentrations in the dorsal anterior cingulate cortex and impulsive decision making [36]. Based on these findings, we infer that the activity of glutamatergic synapses in the ACC may underlie the observed phenomenon. Moreover, the hippocampus and amygdala are important for decision making, asides from their roles in memory encoding and retrieval [37, 38]. Further studies are necessary to determine the brain regions that are involved in food foraging decision-making strategies.

It is well known that many rodents (including rats) have an inherent preference for sweet foods. Standard food represents a low-value reward, whereas sweet food is a high-value reward. In our experiments, rats selected the sweet reward as the optimal choice. In addition, foraged sweet-food pellets were significantly increased compared to standard food pellets under Condition 3, a finding that is consistent with the fact that rats preferred choosing a high-value reward [39]. Manipulations with MK-801, SCH23390, and haloperidol had no effects on foraging decision in the presence of high-value foods, indicating that neither NMDA nor dopamine receptors are involved in modulating value-based decision-making under Condition 3. These results differ from those of previous studies, in which it was reported that dopamine receptors are able to modulate effort-related cost-benefit decision-making in T-maze performance and lever-pressing/feeding choice tasks [26, 40–42]. We posit that the differential results may be due, at least in part, to drugs having divergent effects in different paradigms. Although we did not find evidence of NMDA and dopamine receptors-mediated effects on decision-making, the existence of multiple diverse evaluation circuits in the brain for numerous behavioral strategies cannot be ruled out.

In contrast to the simple scenarios under Conditions 2 and 3, when both high-value food and standard desirable food were presented in the presence of a conspecific under Condition 4, the ratio of foraged standard food pellets in the rat- residing cage did not differ significantly from that of foraged sweet food in the no-conspecific cage. It is reasonable to suppose that there are at least two types of food-foraging decision-making pathways: social-based and value-based [43–45]. Rats traded the preferences of both scenarios and foraged equal amounts of food from both cages. Rats not only made choices for high-value food and a socially desirable environment, but also balanced contextual differences between high-value food and the socially desirable scenario. This circumstance is similar to social context alterations and other multiple contextual factors that are able to modulate both behaviors and signals in task-relevant neural networks [46]. Both MK-801 and SCH23390 disrupted the ability for balancing the preference for foraging high-value food over food from a socially desirable environment, suggesting that NMDA receptors are able to modulate decision-making **vis-à-vis** high-value rewards and socially desirable contexts. We speculate that D1 receptors play roles in modulating the influence of complex (Condition 4), but not simple (Condition 2), social dynamics on food-foraging decisions. Moreover, there was no significant difference between foraged sweet-food pellets and standard food pellets after haloperidol treatment (Condition 4) compared with SCH23390 treatment. This is explained by previous findings, i.e., that D1 and D2 receptors, each with distinct pharmacological profiles, play dissociable roles in the reinforcement of learning underlying decision-making [47].

Entrainment to food regulated by a food-entrainable oscillator (FEO) with circadian properties can mediate food-anticipatory activity in mammals that is characterized by a daily increase in locomotor activity before mealtime when food availability is restricted to a particular time each day. FEOs driving anticipatory rhythms are embedded in hypothalamic circuits responsible for the homeostatic regulation of eating and metabolism [48]. Feeding behavior is thought to be under the control of circulating hormones, including ghrelin, glucagon-like peptide-I, leptin, insulin, as well as central factors, such as neuropeptide Y and agouti-related protein (AgRP), which have been shown to be altered before meal times in a manner that parallels increases in food anticipatory activity [49–54]. These appetite-related factors (i.e. ghrelin, leptin, AgRP and neuropeptide Y) have been reported to moderate overeating/obesity, affecting the anticipation, consumption, and acquisition of food [55]. Indeed, studies have suggested that ghrelin and glucagon-like peptide-I may mediate gut-brain crosstalk and may be involved in the generation of food anticipatory activity, which precedes food foraging [54, 56]. In studies examining the overlapping neural circuitry active in behaviors related to consumption of drugs of abuse and food addiction (excess intake of palatable foods), endocrine signals have been shown to have effects on dopaminergic function in the mesolimbic circuitry, which has involved in the motivation for drug intake and food [57]. Dagher et al. found that circulating peptides are detected by the appetitive network centers around four interconnected brain regions including insula, amygdala, striatum and orbitofrontal cortex [58]. In the light of the evidence indicating that food anticipatory activity can modulate food foraging strategies, further work should be conducted to delineate the roles of FEOs, neuroendocrine hormones, and gut hormones on food foraging decision making, particularly in a socially competitive environment.

## Conclusion

While NMDA receptors play essential roles in decision-making in both social- and value-settings, it is likely that complex neurotransmitters also play roles in influencing decision-making processes in social contexts. In our study, we made use of an animal model to examine the significance of mimetic desire on decision-making. It is our hope that future studies will: (i) provide information on the brain area networks/circuits involved in these processes, such as the ACC, ventromedial prefrontal cortex, medial prefrontal cortex, orbitofrontal cortex, and nucleus accumbens; and (ii) assess potential therapeutic interventions. Such gains in knowledge could revolutionize the diagnosis and treatment of neurological and psychiatric patients with pathological social decision-making difficulties.
